# Understanding Well-Being in the Classroom: A Study on Italian Primary School Teachers Using the JD-R Model

**DOI:** 10.3390/ejihpe15110234

**Published:** 2025-11-15

**Authors:** Maria Francesca Trocino, Giovanni Schettino, Vincenza Capone

**Affiliations:** Department of Humanities, University of Naples Federico II, 80133 Naples, Italy; ma.trocino@studenti.unina.it (M.F.T.); giovanni.schettino@unina.it (G.S.)

**Keywords:** teachers, self-efficacy, well-being, job satisfaction, overload, work autonomy

## Abstract

Teaching has always been recognized as one of the professions with the highest risk of burnout, thus highlighting the need to take into account how organizations could promote a “healthier” work life. Accordingly, literature has gone beyond the conceptualization of well-being as merely the “absence of illness” to concluding that it must be regarded as a state of complete health. The current study adopts this theoretical approach to address the limited literature on factors affecting the well-being of Italian primary school teachers. Specifically, responses to a self-report questionnaire completed by 142 Italian primary school teachers showed that work self-efficacy was positively associated with job satisfaction, which in turn was positively related to well-being. Consequently, job satisfaction fully mediated the relationship between self-efficacy and well-being. Furthermore, both work overload and work autonomy were found to be negatively associated with teacher well-being. These findings can inform the design of targeted interventions aimed at enhancing the performance and psychological health of primary school teachers by managing demands and fostering effective resources.

## 1. Introduction

Understanding the determinants of teachers’ well-being necessitates a comprehensive theoretical framework that includes both risk and protective factors within the workplace. One of the most influential models in this regard is the Job Demands–Resources (JD-R) model (Demerouti et al., 2001; Bakker & Demerouti, 2014). This model posits that every job is characterized by specific job demands and job resources, which jointly influence employees’ health and motivation. More precisely, job demands refer to those physical, psychological, social, or organizational aspects of work that require sustained effort and are therefore associated with physiological or psychological costs (Cooper et al., 2001). Among these, work overload—defined as an excessive amount of tasks or responsibilities requiring sustained cognitive and emotional effort—represents a critical demand that can undermine well-being if not adequately managed (Jomuad et al., 2021). In contrast, job resources are aspects of the job that help achieve work goals, reduce the costs of job demands, and stimulate personal growth and development. Resources can be organizational (e.g., work autonomy), interpersonal (e.g., support from colleagues), or personal (e.g., work self-efficacy, the belief in one’s ability to effectively manage professional challenges) (Morris et al., 2017).

Work autonomy, in particular, has been widely studied as a core organizational resource. Traditionally, it is defined as the degree of discretion and control employees have over how to perform their tasks, including decision making about methods, timing, and priorities (Deci & Ryan, 2000). A substantial body of research links autonomy to positive outcomes, such as higher motivation, job satisfaction, and psychological well-being (Demerouti et al., 2001; Deci & Ryan, 2000). However, recent evidence suggests that its effects are not universally positive (Dettmers & Bredehöft, 2020) but rather contingent upon contextual factors. In supportive environments, autonomy can enhance engagement and buffer against stress, whereas in poorly structured settings, it may impose a decision-making burden, increasing uncertainty and responsibility, which can paradoxically heighten stress and reduce overall well-being (Avanzi et al., 2018; Karasek, 1979; Maslach & Leiter, 2008). These findings underscore the complexity of autonomy as a resource and the need to examine its interaction with other job characteristics and personal resources.

According to the JD-R model, two parallel processes operate: a health-impairment process, whereby excessive demands exhaust employees’ energy and lead to strain, and a motivational process, whereby resources foster engagement, job satisfaction, and well-being. When demands chronically outweigh resources, the health-impairment pathway becomes dominant, increasing the risk of stress-related outcomes such as burnout (Bakker & Demerouti, 2014; Demerouti et al., 2001).

Burnout represents one of the most severe consequences of prolonged exposure to high job demands in the absence of adequate resources (Leiter, 1992). It is defined as a psychological syndrome resulting from chronic, unmanaged work-related stress and is characterized by three dimensions: emotional exhaustion, reflecting the depletion of emotional resources; depersonalization or cynicism, involving a detached or negative attitude toward work and its recipients; and reduced professional accomplishment, indicating a sense of ineffectiveness and lack of achievement (Maslach et al., 2001). Teachers are particularly vulnerable to burnout because their role requires sustained cognitive and emotional investment (Woolfolk et al., 1990), frequent interpersonal interactions, and high accountability for student outcomes (Maslach et al., 2001; Mijakoski et al., 2022; Scarzello & Prino, 2025). These conditions can erode personal resources and compromise both psychological health and professional performance, with detrimental effects on student learning and organizational functioning (Pakdee et al., 2025). Consequently, identifying the mechanisms that mitigate burnout and promote well-being is a priority for educational systems.

Regarding this fundamental outcome, research has shifted in recent years from an approach that focuses solely on preventing negative outcomes to a strength-based perspective that emphasizes the promotion of positive mental health. In this context, Keyes (2002) model provides a comprehensive framework by conceptualizing mental health as a continuum ranging from languishing to flourishing. Flourishing is defined by the presence of positive emotions and optimal functioning across three domains: emotional well-being (e.g., life satisfaction, positive affect), psychological well-being (e.g., autonomy, personal growth, purpose in life, positive relationships), and social well-being (e.g., social integration, acceptance, and contribution) (Keyes & Waterman, 2003). This multidimensional view underscores that well-being is not merely the absence of illness but a state of positive functioning, which is essential for teachers’ effectiveness, resilience, and quality of educational outcomes.

### The Current Study

This study adopts a positive psychology perspective within the JD-R framework to investigate the factors affecting the mental well-being of Italian primary school teachers. Consistent with the main assumption of the JD-R model (Demerouti et al., 2001; Bakker & Demerouti, 2014), we expected that job demand, specifically work overload, would be negatively associated with both job satisfaction and mental well-being. Conversely, on the basis of evidence of its protective role (Donohoo, 2018; Granziera & Perera, 2019; Harmsen et al., 2018; Skaalvik & Skaalvik, 2007, 2010), we hypothesized that the personal resource of work self-efficacy (Bandura, 1997, 2000) would be positively associated with these same outcomes. Self-efficacy has also been linked to inclusion practices (Wray et al., 2022) and to teachers’ engagement and well-being (Shu, 2022). Based on mixed results regarding work autonomy (Avanzi et al., 2018; Karasek, 1979), we also investigated its role in affecting teachers’ well-being, hypothesizing a positive association. Moreover, given that job satisfaction is a key indicator of occupational well-being influenced by both demands and resources (Caprara et al., 2006), we propose a mediating role for job satisfaction in these relationships. Finally, we estimated the prevalence of mental health among participants.

**Figure 1:**
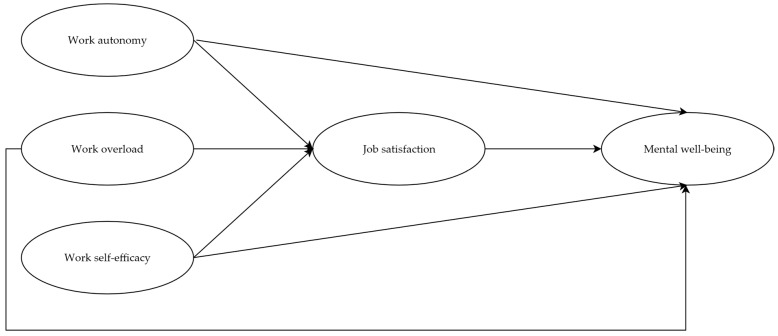
Research hypotheses.

Hypotheses tested are reported below, as well as represented in Figure 1:

**Hypothesis 1** **(H1):** *Work autonomy is positively associated with job satisfaction (H1a) and mental well-being (H1b).*

**Hypothesis 2** **(H2):** *Work overload is negatively associated with job satisfaction (H2a) and mental well-being (H2b).*

**Hypothesis 3** **(H3):** *Work self-efficacy is positively associated with job satisfaction (H3a) and mental well-being (H3b).*

**Hypothesis 4** **(H4):** *Job satisfaction mediates relationships between work autonomy (H4a), work overload (H4b), work self-efficacy (H4c), and mental well-being.*

## 2. Materials and Methods

### 2.1. Participants and Procedure

The current study used a cross-sectional design. A non-probability convenience sampling method was employed to recruit participants. Specifically, they were recruited by advertising a link to a self-report questionnaire on some of the main Italian social networking sites (e.g., Facebook groups), specifically on closed social media groups for Italian primary school teachers.

The inclusion criterion was that the participants were Italian primary school teachers. Participants were informed about the anonymity of the data collection and signed the informed consent form. Thereafter, they completed an online self-report questionnaire implemented on the Google Forms platform. The questionnaire took approximately 15 min to complete and required a mandatory response to each item, so no respondents had missing values.

Among the invited participants, a total of 150 Italian individuals met the inclusion criteria and completed the questionnaire. Of these, only 8 participants declared to be employed in private primary schools, leading us to exclude them from the analyses. Therefore, among the 142 primary school teachers from public schools, the majority were female (94.4%)—which is consistent with the gender distribution of the Italian primary teacher population as affirmed by the Gender Data Portal (2022)– and aged between 23 and 66 years (M = 46.91; SD = 10.41). Additionally, the participants reported an average of 16.81 years of professional experience (SD = 12.18) and an average organizational tenure of 9.79 years (SD = 17.04). Furthermore, 59.2% of the participants were from southern Italy, and 70.4% were married.

Data were collected between February 2023 and July 2023. All procedures followed were in accordance with the Helsinki Declaration and the General Data Protection Regulation. Participation was anonymous, no incentive was given, and informed consent was obtained from all participants. The respondents were allowed to withdraw from the study at any time.

### 2.2. Measures

The questionnaire included an initial screening question requiring participants to confirm their current employment status, followed by an informed consent form. Subsequently, the following measures were administered in the same order to all participants.

*Work autonomy* was assessed using five items from the Job autonomy subscale of the Majer-D’Amato Organizational Questionnaire 10 (D’Amato & Majer, 2005). The instrument evaluates the perceived job autonomy individuals experience during their work tasks through a 5-point scale ranging from 1 = “false” to 5 = “true” (e.g., “In my work, I have a certain degree of autonomy”). Cronbach’s α for the current study = 0.79.

*Work overload* was assessed using the 4-item scale by Moore (2000). The instrument evaluates the perceived work overload through a 7-point Likert scale ranging from 1 = “Strongly disagree” to 7 = “Strongly agree”. An example item is: “I feel that the number of requests, problems, or tasks I deal with is more than expected.” Cronbach’s α = 0.89.

*Work self-efficacy* was assessed using the 6-item Work-Efficacy Scale (Borgogni et al., 2001). The instrument evaluated workers’ beliefs in their ability to effectively manage tasks and challenges related to their professional role. Each item (e.g., “I am always able to master the emergencies and unexpected events related to my work”) was rated on a 7-point scale ranging from “strongly disagree” (1) to “strongly agree” (7). Cronbach’s α for the current study = 0.81.

*Job satisfaction* was assessed using 1 item (i.e., “What is your level of satisfaction with your job?”) as suggested by Cortese and Quaglino (2006). Higher scores indicate greater satisfaction with one’s own job. The item was rated on a 7-point scale ranging from “I am extremely dissatisfied” (1) to “I am extremely satisfied” (7).

*Mental well-being* was assessed using the Italian Mental Health Continuum Short Form (MHC-SF) (Petrillo et al., 2015). The instrument included 14 items (e.g., “During the past month, how often did you feel happy?”, “During the past month, how often did you feel that you belonged to a community?”, “During the past month, how often did you feel that you had warm and trusting relationships with others”) evaluated on a 6-point scale ranging from 0 = never to 5 = every day. To test the hypothesized model of the study, the total score of the MHC-SF was used as a single indicator of participants’ mental well-being. Cronbach’s α for the current study = 0.95.

### 2.3. Statistical Analyses

Statistical analyses were performed in order to evaluate the internal reliability of the instruments adopted in this study. Additionally, to assess potential differences across psychosocial variables according to three groups: flourishing, moderately mentally healthy, and languishing, the three main ANOVA assumptions were evaluated (i.e., normality of the distribution, homogeneity of variance, and independence of observations) (Barbaranelli, 2006). In order to evaluate our hypotheses, we adopted a reflective partial least squares-structural equation modeling (Ringle et al., 2015, 2024). This technique was chosen for its established robustness in providing reliable estimates, particularly with small sample sizes and non-normally distributed data, as documented by Hair et al. (2019). PLS-SEM consists of a measurement (outer) model and a structural (inner) model. The former model assesses the relationships between latent variables and their indicators, whereas the latter examines the relationships among latent constructs (Hair et al., 2019). Significance levels for both the outer and inner model parameters were determined through bootstrapping with 5000 subsamples, using a percentile bootstrap for confidence intervals and a random seed for the random number generator (Ringle et al., 2015, 2024). Once the measurement model was specified, its adequacy was ascertained by examining the following criteria: factor loadings > 0.5, Cronbach’s alpha > 0.7, and Rho A > 0.7 (indicator reliability). Additionally, the convergent and discriminant validity of the constructs were assessed. Specifically, the former type of validity was verified by examining the average variance extracted (AVE) of the construct, which should be equal to or greater than 0.5 (Hair et al., 2017). Discriminant validity was assessed using the Fornell–Larcker criterion, which consists of comparing the square root of the AVE with the correlation between latent constructs (Venturini & Mehmetoglu, 2019). The evaluation of the structural model was based on the path coefficient values and their statistical significance (Venturini & Mehmetoglu, 2019).

## 3. Results

### 3.1. Descriptive Statistics

Table 1 presents the descriptive statistics (mean, standard deviation) for each item in the evaluated model. The item means range from 1.67 (WELL.12) to 6.16 (S_EFF.3). Notably, the highest mean values pertain to items assessing participants’ perceived ability to manage their work-related tasks effectively. Adopting the classification proposed by Keyes (2002), which is based on specific components of mental well-being, the majority of participants (47.89%) were identified as flourishing, 39.43% as moderately mentally healthy, and 12.68% as languishing. Additionally, since the assumption of normality was not consistently met across all variables and groups (i.e., flourishing, moderately healthy, and languishing), and the assumption of homogeneity of variances was violated for several variables, the non-parametric Kruskal–Wallis test was adopted to examine differences in the levels of the variables considered across three groups: flourishing (1), moderately mentally healthy (2), and languishing (3). The degrees of freedom (*df*) for the analysis were 2, determined by the formula *df* = *k* − 1, where k represents the total number of groups compared (i.e., flourishing, moderately mentally healthy, and languishing). The results revealed statistically significant differences between the groups for all these variables (all *p* values < 0.05). Regarding work autonomy, a significant difference emerged (H = 7.624, *p* = 0.022, *η*^2^ = 0.04): mean ranks for work autonomy for the flourishing group = 61.81; for the moderately mentally healthy group = 78.84; for the languishing group = 85.28, suggesting that the languishing group reported the highest levels of work autonomy whereas the flourishing group reported the lowest. Furthermore, a significant difference emerged in work overload (H = 11.623, *p* = 0.003, *η*^2^ = 0.07): mean ranks for the flourishing group = 62.60; for the moderately mentally healthy group = 73.33; for the languishing group = 99.42. Consistent with these results, a significant difference was observed in work self-efficacy levels across the groups (H = 9.159, *p* = 0.010, *η*^2^ = 0.05): mean ranks for the flourishing group = 82.31; for the moderately mentally healthy group = 60.77; for the languishing group = 64.06. Similarly, statistically significant differences were also found concerning job satisfaction (H = 25.345, *p* < 0.001, *η*^2^ = 0.15): mean ranks for job satisfaction for the flourishing group = 86.60, for the moderately mentally healthy group = 62.97, for the languishing group = 41.00. Hence, the flourishing group presented the highest level of job satisfaction, followed by the moderately mentally healthy group, and the languishing group reported the lowest level.

### 3.2. The Measurement Model

Table 1 displays the results of the measurement model, showing strong relationships between the latent constructs and items with factor loadings > 0.50, ranging from 0.51 to 0.89 (Table 1). Internal consistency (Cronbach’s α and rho_A) and average variance extracted (AVE) for all latent constructs were above the minimum threshold values. The results of the Fornell–Larcker criterion (Table A1) indicated that the discriminant validity of the constructs was established. As Podsakoff et al. (2003) suggested, we examined the potential presence of common method bias in the data and found no correlation greater than the 0.90 threshold (Bagozzi et al., 1991; Kock, 2015). Furthermore, the Harman one-factor test indicated that one factor accounted for 33.54% of the covariance. Since the value was below the 50% threshold, it can be stated that common method bias was not a concern in this study. Collinearity statistics were also examined (Table A2), with all the predictors showing VIF values below 3.3 (Hair et al., 2019), indicating no significant multicollinearity issues.

### 3.3. The Structural Model

**Figure 2:**
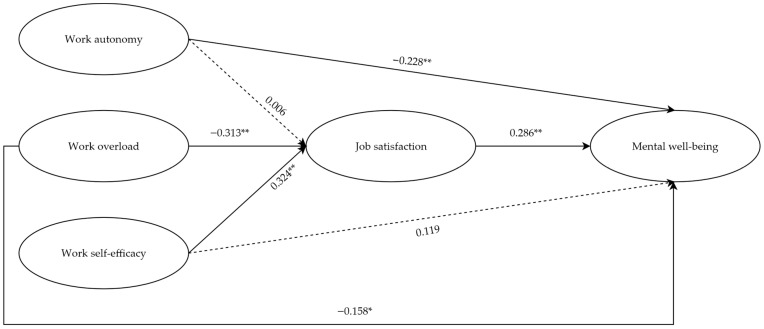
Structural model with standardized regression coefficients. **Notes**. ** *p* < 0.01, * *p* < 0.05. Significant relationships are marked by solid lines, and non-significant relationships are marked by dashed lines.

Since a satisfactory measurement model was ascertained, hypotheses were formally evaluated with the structural model of PLS-SEM (Figure 2). Findings confirmed almost all the hypotheses (Table 2).

Specifically, in line with H2a and H2b, the expected negative associations between work overload and both job satisfaction and mental well-being were confirmed, highlighting the detrimental effect of excessive demands on these outcomes. Additionally, H3a was confirmed, whereas H3b was not supported.

However, contrary to expectations, H1a—which suggested a direct positive association between work autonomy and job satisfaction—was not confirmed since results indicated no significant relationship. Similarly, H1b was not supported. More precisely, although statistically significant, the association between work autonomy and mental well-being was negative.

Finally, regarding the variance explained, it is 23.3% of the variance in job satisfaction and 27.1% in mental well-being.

Mediation analyses were run to verify the hypothesized mediating effects of job satisfaction. Specifically, analyses revealed a full mediation of job satisfaction on the relationship between work self-efficacy and mental well-being and a partial mediating effect on the path between work overload and mental well-being. Conversely, job satisfaction did not mediate the path between work autonomy and mental well-being.

## 4. Discussion

The purpose of this study was to investigate the factors that could promote the well-being of primary school teachers in the Italian context, with the broader aim of contributing to their mental health and, consequently, enhancing their work performance.

In line with our expectations, the findings revealed a positive association between participants’ job satisfaction and mental well-being. This result aligns with the organizational literature, which indicates that job satisfaction and well-being are strongly associated (Avanzi et al., 2018).

However, in contrast to this path and our hypotheses, a negative association emerged between work autonomy and well-being. Regarding this result, it contrasts with the literature, which has widely emphasized the positive role of work autonomy, which is often associated with higher job satisfaction, representing one aspect of well-being, and with increased motivation among workers (Hackman & Oldham, 1976). Work autonomy is also considered a protective factor against the negative effects of job demands, such as workload (Demerouti et al., 2001). Additionally, Deci and Ryan (2000) describe work autonomy as a psychological need and a crucial resource for achieving a higher level of well-being. A possible explanation for the unexpected result in our study could be found by assuming a more complex relationship between work autonomy and mental health. It is plausible to assume that work autonomy may create decision-making difficulties and an excessive sense of personal responsibility, which could result in higher stress and reduced well-being (Avanzi et al., 2018). Specifically, in the school workplace, teachers who lack good social support in their work, such as when managing students’ behavior, are more likely to experience a decrease in their well-being (Avanzi et al., 2018; Nicolosi et al., 2023). In support of this reasoning, the Job Demand-Control model (Karasek, 1979) suggests that when both work autonomy and job demands are high, workers may experience stress. This is also consistent with the TALIS data, as this report (2018) indicates that, in Italy, teachers have a high degree of decision-making autonomy, for example, in the content of courses or in selecting teaching materials. However, such responsibilities are not always supported by adequate organizational resources, such as professional feedback (OECD, 2018). The combination of high work autonomy and limited organizational support reflects a context in which decision-making autonomy can become a potential source of isolation and overload rather than a resource for well-being (Maslach & Leiter, 2008).

With respect to work overload, the findings of our study are consistent with the JD-R model and evidence reporting that this job demand can erode work–life balance and fuel emotional exhaustion (Madigan & Kim, 2021), particularly in a non-supportive climate (Avanzi et al., 2018; Bowling et al., 2015; Dicke et al., 2018). Indeed, workers who experience an excessive workload may struggle to maintain a healthy work–life balance and are more likely to experience emotional exhaustion (Madigan & Kim, 2021).

In contrast to the existing literature, we did not observe a significant direct association between work self-efficacy and mental well-being (Borgogni et al., 2009; Caprara et al., 2003; Hargreaves, 2021; Judge et al., 2000; Judge & Bono, 2001; Luthans et al., 2006). However—and in line with prior evidence—higher self-efficacy was positively associated with greater job satisfaction, plausibly because teachers who perceive themselves as effective at work tend to deploy more adaptive coping strategies and therefore report higher well-being (Brief, 1998; Spector, 1997) and lower exposure to stressors (Brief, 1998). Consistent with this view, the mediating role of job satisfaction in the relationship between work self-efficacy and mental well-being in our sample reinforces the thesis that satisfaction is a proximal indicator of work-related well-being (Capone et al., 2019; Capone & Petrillo, 2020).

Finally, following Keyes’s classification (Keyes, 2002; Keyes & Waterman, 2003), most participants were flourishing or moderately mentally healthy, with a smaller languishing subgroup. Consistent with the group comparisons, languishing teachers reported the highest workload and work autonomy, which could explain the particularly low job satisfaction observed in this group. Prior studies have shown that teachers can shift between flourishing and non-flourishing states over time (Skaalvik & Skaalvik, 2020) and that such shifts are associated with intentions to leave the job. Therefore, these profiles should be monitored longitudinally (Rothmann & Redelinghuys, 2020). Together, these findings underscore the need for systemic strategies to promote teachers’ mental health and professional satisfaction in primary education. However, systematic reviews underline the scarcity of structured interventions (Cann et al., 2023).

### Limitations and Suggestions for Future Research

We acknowledge that our study has certain limitations that need to be considered. First, the study employed a cross-sectional design, which does not allow for inferring cause-and-effect relationships among the variables. Second, the study employed a non-probability convenience sampling method, recruiting participants through social media. This approach may have introduced a self-selection bias, as individuals who chose to participate might have specific characteristics (e.g., higher digital skills) or a higher interest in the topic compared to the general population. Consequently, based on this consideration, the findings may not be generalizable to the broader population of Italian teachers. Third, the sample size adopted may have limited the statistical power to detect weaker effects or conduct more complex subgroup analyses. Considering these limitations, future research would benefit from adopting a longitudinal or experimental design to examine the causal relationships between work autonomy, work overload, self-efficacy, job satisfaction, and well-being. Moreover, using larger, probabilistic samples could ensure the generalizability of the findings. Additionally, it would be interesting to investigate the role of specific teacher efficacy beliefs (e.g., efficacy in classroom management, efficacy in technology adoption) and further organizational factors (e.g., organizational climate, co-worker support) that may affect teachers’ job satisfaction.

## 5. Conclusions

Our results underscore the need to develop interventions that increase teachers’ job satisfaction through job design oriented toward reducing work overload (Daniels et al., 2017). Specifically, this implies redistributing tasks, streamlining administrative procedures, balancing class and duty assignments, and clarifying priorities so that effort aligns with core teaching activities. Alongside workload reallocation, ensuring adequate resources is essential—e.g., timely access to teaching materials and digital tools, the availability of support staff, and realistic schedules that prevent the spillover of tasks outside contracted hours. Professional development programs that strengthen professionals’ skills—both technical and hard (Schettino & Capone, 2025; Schettino et al., 2024)—should be structured with practice-oriented modules, coaching/mentoring, and opportunities for feedback, thereby fostering job competence, perceived efficacy, and, in turn, job satisfaction and mental well-being. Finally, the negative relationships between work autonomy and well-being underscore the importance of pairing autonomy with adequate organizational scaffolding, including clear task boundaries, explicit decision-making guidelines, regular supervisory and peer feedback, and accessible collegial support. Framing autonomy within these guardrails can prevent it from turning into isolation or role overload, thus safeguarding the mental health of primary school teachers and, ultimately, their performance.

## Figures and Tables

**Table 1:** Factor loadings, Cronbach’s α, Rho A, and AVE of the measurement model.

Item	Mean	Std. Dev	Range	W_AUTO	OVER	S_EFF	WELL
W_AUTO.1	2.68	1.38	1–5	0.807			
W_AUTO.2	2.70	1.34	1–5	0.837			
W_AUTO.3	2.62	1.32	1–5	0.510			
W_AUTO.4	2.74	1.34	1–5	0.823			
W_AUTO.5	2.80	1.32	1–5	0.646			
OVER.1	5.25	1.62	1–7		0.853		
OVER.2	4.25	1.84	1–7		0.864		
OVER.3	5.21	1.67	1–7		0.846		
OVER.4	4.66	1.92	1–7		0.892		
S_EFF.1	5.70	1.10	1–7			0.660	
S_EFF.2	5.47	1.35	1–7			0.689	
S_EFF.3	6.16	0.77	1–7			0.668	
S_EFF.4	5.66	1.11	1–7			0.738	
S_EFF.5	5.71	0.92	1–7			0.725	
S_EFF.6	5.75	0.89	1–7			0.796	
WELL.1	2.97	1.29	0–5				0.840
WELL.2	3.68	1.41	0–5				0.885
WELL.3	3.16	1.27	0–5				0.881
WELL.4	3.10	1.41	0–5				0.747
WELL.5	3.27	1.28	0–5				0.849
WELL.6	3.36	1.33	0–5				0.767
WELL.7	3.59	1.38	0–5				0.803
WELL.8	3.49	1.36	0–5				0.772
WELL.9	3.70	1.49	0–5				0.857
WELL.10	3.22	1.56	0–5				0.814
WELL.11	3.12	1.64	0–5				0.821
WELL.12	1.67	1.55	0–5				0.699
WELL.13	2.04	1.44	0–5				0.655
WELL.14	1.84	1.51	0–5				0.692
*Cronbach’s α*				0.787	0.888	0.807	0.953
*Rho A*				0.843	0.911	0.810	0.959
*AVE*				0.541	0.746	0.510	0.623

Notes. W_AUTO = work autonomy; OVER = work overload; S_EFF = work self-efficacy; WELL = mental well-being; Std. dev = standard deviation; Rho A = composite reliability; AVE = average variance extracted.

**Table 2:** PLS-SEM results for hypothesis testing.

	Path Coefficients	SE	CI
*Direct effects*			
W_AUTO → J_SAT	0.006	0.073	(−0.125, 0.166)
OVER → J_SAT	−0.313 **	0.093	(−0.471, −0.111)
S_EFF → J_SAT	0.324 **	0.097	(0.111, 0.492)
W_AUTO → WELL	−0.228 **	0.067	(−0.346, −0.080)
OVER → WELL	−0.158 *	0.079	(−0.302, −0.003)
S_EFF → WELL	0.119	0.085	(−0.053, 0.279)
J_SAT → WELL	0.286 **	0.104	(0.069, 0.472)
*Indirect effects*			
W_AUTO → J_SAT → WELL	0.002	0.021	(−0.040, 0.043)
OVER → J_SAT → WELL	−0.090 *	0.036	(−0.182, −0.032)
S_EFF → J_SAT → WELL	0.092 *	0.040	(0.031, 0.194)
*Total effects*			
W_AUTO → WELL	−0.226 **	0.065	(−0.335, −0.074)
OVER → WELL	−0.247 **	0.075	(−0.384, −0.086)
S_EFF → WELL	0.211 **	0.078	(0.051, 0.353)

**Notes.** W_AUTO = work autonomy; OVER = work overload; S_EFF = work self-efficacy; J_SAT = job satisfaction; WELL = mental well-being; SE = standardized error; CI = confidence interval; * *p* < 0.05, ** *p* < 0.01.

## Data Availability

The dataset that supports the findings of this study is available from the corresponding author upon reasonable request.

## Appendix A

**Table A1:** Discriminant validity with the Fornell–Larcker criterion.

	W_AUTO	OVER	S_EFF	J_SAT	WELL
**W_AUTO**	**0.736**				
**OVER**	0.221	**0.864**			
**S_EFF**	−0.158	−0.108	**0.714**		
**J_SAT**	−0.115	−0.347	0.356	**-**	
**WELL**	−0.314	−0.320	0.274	0.409	**0.789**

**Notes.** W_AUTO = work autonomy; OVER = work overload; S_EFF = work self-efficacy; J_SAT = job satisfaction; WELL = mental well-being. Values in bold on the diagonal represent the square root of the Average Variance Extracted (AVE). Discriminant validity is established as each diagonal value is higher than all other correlations in the corresponding row and column.

**Table A2:** Structural model—Multicollinearity check (Variance Inflation Factors—VIFs).

	J_SAT	WELL
**J_SAT**		1.288
**W_AUTO**	1.072	1.072
**OVER**	1.057	1.184
**S_EFF**	1.032	1.166

**Notes.** W_AUTO = work autonomy; OVER = work overload; S_EFF = work self-efficacy; J_SAT = job satisfaction; WELL = mental well-being.
